# Intravesical Gemcitabine versus Intravesical Bacillus Calmette–Guérin for the Treatment of Non-Muscle Invasive Bladder Cancer: An Evaluation of Efficacy and Toxicity

**DOI:** 10.3389/fonc.2017.00260

**Published:** 2017-11-02

**Authors:** Thiru Prasanna, Paul Craft, Gayathri Balasingam, Hodo Haxhimolla, Ganes Pranavan

**Affiliations:** ^1^Department of Medical Oncology, The Canberra Hospital, Canberra, ACT, Australia; ^2^Australian National University, Canberra, ACT, Australia; ^3^Department of General Medicine, The Calvary Hospital, Canberra, ACT, Australia; ^4^Department of Urology, The Canberra Hospital, Canberra, ACT, Australia

**Keywords:** bladder cancer, carcinoma *in situ*, cystitis, gemcitabine, intravesical therapy

## Abstract

**Background:**

Intravesical Bacillus Calmette–Guérin (BCG) remains the standard adjuvant treatment for non-muscle invasive bladder cancer (NMIBC) following transurethral resection; however, BCG failure and related toxicities are common.

**Objectives:**

To compare the efficacy and toxicity of intravesical BCG and gemcitabine in the treatment of NMIBC.

**Methods:**

Retrospective data were collected in the region of Canberra, Australia from January 2010 to December 2015. The survival cutoff was December 2016. Primary end point was disease-free survival (DFS) and secondary end point was toxicity. After optimal transurethral resection all patients received weekly intravesical BCG or gemcitabine for 6 weeks and maintenance treatment according to their risk. The recurrence was defined as histology proven tumor recurrence (any grade), or appearance of carcinoma *in situ*.

**Results:**

One hundred and three patients were evaluable, 52 treated with BCG and 51 with gemcitabine with a median age of 77 and 78, and were mostly male. Approximately half of each received maintenance therapy. The groups were well balanced, apart from some difference in cancer risk groups. Twenty-one percent in the BCG group and 29% in the gemcitabine group had received prior BCG. Median follow up was 15.0 months. Median DFS was 19.6 months for BCG, whereas median DFS was not reached with gemcitabine. There was a trend toward improved DFS with gemcitabine in multivariate analysis, HR: 0.49 (95% CI: 0.22–1.06, *p* = 0.07). Adverse events were significantly less frequent with gemcitabine (7 versus 44%, *p* ≤ 0.05). There were four cases of systemic BCG infection.

**Conclusion:**

Intravesical gemcitabine was associated with a trend toward better DFS with significantly lower toxicity when compared with BCG. Intravesical BCG remains the standard first-line adjuvant therapy; however, intravesical gemcitabine could be a reasonable alternative in cases where BCG is contraindicated and for patients who are intolerant or refractory to BCG. A prospective phase 3 trial is needed to confirm the benefits of gemcitabine over BCG.

## Introduction

Bladder cancer ranks ninth in international cancer incidence and represents the fourth most common cancer among men in western nations (1). Urothelial cancer of the bladder is more common in older persons, with more than 90% of cases occurring in patients aged 55 years and above (1). At initial presentation, approximately 70% of bladder cancers are non-muscle invasive tumors (NMIBC), which include the entities of carcinoma *in situ* (CIS) and papillary carcinomas of stage Ta and T1 (2, 3).

The mainstay of treatment for non-muscle invasive bladder cancer (NMIBC) is transurethral resection of bladder tumor (TURBT) (4). NMIBC cancer has a recurrence rate of 70%, with 20% of recurrences progressing to advanced disease (3). In light of this, TURBT is commonly followed by local treatment with either intravesical chemotherapy or immunotherapy (3, 4).

Intravesical Bacillus Calmette–Guérin (BCG) has been used post-TURBT since the 1970s; however, about 30% of patients develop recurrence despite this therapy. BCG administration can result in a range of toxicities, including cystitis and more significantly, systemic BCG infection (3). In addition, there is still no consensus surrounding the appropriate duration of maintenance therapy following an initial 6 week induction cycle (5).

Many other chemotherapeutic agents like mitomycin C (MMC), gemcitabine, and epirubicin have been used as intravesical adjuvant therapy post-TURBT as an alternative to BCG or as second-line therapy. Intravesical gemcitabine has been investigated as a potential treatment for NMIBC (6). Gemcitabine is an antimetabolite, which has activity in the treatment of metastatic bladder cancer. With many small studies (7–9) showing good responses in NMIBC with gemcitabine, a randomized controlled study by Addeo et al. found gemcitabine to be superior to MMC in efficacy and less toxic compared to MMC (3). A Cochrane review in 2012 found gemcitabine had similar efficacy to BCG at least in intermediate risk group and superior in BCG refractory patients (10). Though there are multiple single arm studies and a single phase 2 trial compared gemcitabine to BCG (11), there are no head to head randomized phase 3 trials available. Many patients were treated with first-line intravesical gemcitabine in Canberra due to nationwide shortage of BCG around 2010. We conducted a retrospective study to assess the effectiveness and toxicity of intravesical gemcitabine and BCG.

## Materials and Methods

The aim of the study was to compare the effectiveness, as indicated by disease-free survival (DFS) time and toxicity, of intravesical BCG immunotherapy with intravesical gemcitabine chemotherapy in the treatment of patients with NMIBC. This was a retrospective study of sequential patients with CIS, pTa, and pT1 cancers who were treated at in a regional cancer center and a neighboring hospital during the period January 2010–December 2015, including both new diagnoses and recurrent cancers. Survival data cutoff was December 2016. Patients with history prior NMIBC, who have received intravesical BCG more than 12 months ago were included.

Ethics approval was obtained from the ACT Health Human Research Ethics Committee (ACTH-HREC), which is constituted according to National Health and Medical Research Council guidelines and the National Statement on Ethical Conduct in Human Research 2007. The relevant data were collected from medical records, pharmacy records, pathology records, and operative notes.

All patients had undergone initial TURBT and confirmed cancer free with subsequent second cystoscope and biopsy before commencing intravesical therapy. All patients were treated with initial weekly intravesical induction therapy with either BCG or gemcitabine for 6 weeks. Oncotice BCG was instilled at a dose of 500 million colony forming units per treatment with 2 h of retention time, while the other cohort received 2,000 mg of intravesical gemcitabine. Patients received maintenance treatment depending on their recurrence risk profile, provided there was no evidence of recurrence on subsequent cystoscopies. Initial cystoscopy was undertaken approximately 4–6 weeks after the end of induction therapy, and subsequent cystoscopies were at 4 to 6 monthly intervals approximately.

The primary end point of the study was DFS, defined as time from the commencement of induction treatment to recurrence. All recurrences were confirmed by cystoscopic guided biopsy and histology from two standard pathology laboratories in Canberra. The European Association of Urology (EAU) risk stratification scoring system (12) was used to stratify into three groups low (1–4), intermediate (5–9), and high risk (10–17). Recurrence was defined as histology proven tumor recurrence (any grade) or appearance of CIS. Secondary analysis included toxicity evaluation.

### Statistical Method

Survival curves were generated using the method of Kaplan–Meier and a Cox proportional hazards model was used to analyze the association of treatment with DFS. A multivariable analysis was performed, adjusting the treatment effect for tumor grade, number of lesions, EAU risk group and prior BCG therapy. Fisher’s Exact test was used to compare the patients’ baseline characteristics and toxicity. A *p*-value < 0.05 was considered statistically significant. Analyses were conducted using Stata v14.

## Results

A total of 154 patients who received either BCG immunotherapy or gemcitabine chemotherapy for the treatments of NMIBC between 2010 and 2015 were identified. Reliable outcome measures and toxicity data were only available for 103 patients, including 52 treated with BCG and 51 with gemcitabine; these cases were included in the study. All patients have undergone TURBT and single instillation of either MMC or epirubicin in the peri-operative period. A second cystoscopy was performed in all the patients included in this study to confirm complete resection of the tumor.

Patient characteristics were similar in both groups (see Table 1). Most patients were male in both groups with a median age of 77 and 78 in BCG and gemcitabine, respectively. Most patients in both groups had a single identified tumor, majority being high-grade 58 and 60% in BCG and gemcitabine arm, respectively; while others being either CIS or low grade. Twenty-one percent in BCG group and 29% in gemcitabine group had received prior BCG. Tumor characteristics and prior history of recurrence were used to stratify patients into EAU risk groups. Twenty-five percent in BCG group and 15% in gemcitabine group had high risk of recurrence, while 4% BCG and 19% of gemcitabine patients had low risk of recurrence. The average time for induction treatment from the time of surgery was 4–6 weeks in both groups. The intended retention time according to local protocol was 2 h for BCG and 1 h for gemcitabine, with 85% of the patients in BCG and 90% of patients in gemcitabine achieving this. The total duration of treatment was 6 weeks. The low risk group did not receive maintenance therapy while intermediate and high-risk group received maintenance therapy as per standard guidelines. Twenty-nine out of 53 patients in the BCG group and 27 out of 51 in gemcitabine group proceeded to maintenance treatment.

**Table 1:** Patient characteristics.

Patient characteristics	BCG (52)	GEM (51)	*p*-Value
Median age	77	78	
Sex, *n* (%)			
Male	43(83%)	43(84%)	0.8
Female	9(17%)	8(16%)	
Histology			
High grade	30(58%)	30(60%)	0.9
Low grade	07(13%)	08(15%)	
Carcinoma *in situ*	15(29%)	13(25%)	
Number of tumor foci			
Single	38(73%)	35(69%)	0.6
Multiple	14(27%)	16(31%)	
European association of urology risk group			
Low	2(4%)	10(19%)	0.02
Intermediate	37(71%)	34(66%)	
High	13(25%)	7(15%)	
Prior BCG therapy	11(21%)	15(29%)	0.3
Maintenance therapy	29(56%)	27(53%)	0.7

*GEM, intravesical gemcitabine; BCG, intravesical Bacillus Calmette–Guérin*.

### Effectiveness

**Figure 1:**
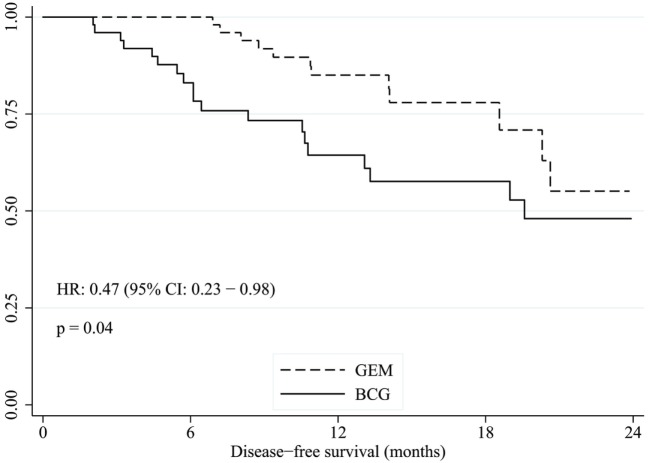
Kaplan–Meier estimate of disease-free survival time with gemcitabine versus BCG. GEM, intravesical gemcitabine; BCG, intravesical bacillus Calmette–Guérin.

The median duration of follow up was 15.0 months. The median DFS was approximately 19.6 months for BCG, whereas median DFS was not reached with gemcitabine, with an unadjusted HR of 0.47 (95% CI: 0.23–0.98, *p* = 0.04) favoring gemcitabine (Figure 1). After adjusting for grade, number of lesions (>1 versus 0.1), EAU risk, and prior BCG, the treatment effect was no longer statistically significant (HR: 0.49, 95% CI: 0.22–1.06, *p* = 0.07, Table 2). The 2-year DFS rates were numerically higher in the GEM arm (55.1 versus 48.0%) but this was non-significant (*p* = 0.32). The DFS rate at 6 and 12 months were consistently better with gemcitabine compared to BCG (100 versus 83% and 85 versus 64%, respectively). Two patients in BCG group and one patient in gemcitabine group progressed to higher stage tumors and underwent cystectomy.

**Table 2:** Cox proportional hazard models.

	Multivariable	
	HR (95% CI)	*p*-Value
GEM versus BCG	0.49 (0.22, 1.06)	0.07
Grade		
CIS^a^		
Low	0.52 (0.21, 1.31)	0.04
High	1.90 (0.60, 6.04)	
Number of lesions		
1	1.01 (0.41, 2.49)	0.97
>1		
Risk		
High^a^		
Intermediate	0.56 (0.13, 2.53)	0.04
Low	0.30 (0.11, 0.78)	
Prior BCG		
No	0.67 (0.28, 1.61)	0.37
Yes		

*GEM, intravesical gemcitabine; CIS, carcinoma in situ; BCG, intravesical bacillus Calmette–Guérin*.

*^a^Referenced category*.

### Toxicity Evaluation

Eight patients from the BCG group discontinued treatment due to treatment related side effects. Four of these patients discontinued due to systemic BCG infection and were treated with an antituberculous antibiotic regimen and subsequently went onto continue with intravesical gemcitabine. Fifty percent of patients receiving BCG had at least 1–2 weeks of treatment delay due to toxicity whereas only 10% had treatment interruption with gemcitabine.

Other common side effects in both arms were chemical cystitis, urosepsis, suprapubic discomfort, hematuria, and urinary frequency (see Table 3). Overall 44% of patients experienced some form of side effects with BCG, whereas only 7% experienced side effects with gemcitabine; these were mainly chemical cystitis and supra pubic discomfort. Fifteen patients who received BCG developed symptomatic cystitis, with five having culture proven urinary sepsis. Another ten patients were treated with antibiotics, for whom cultures were either negative or not available. Eight patients discontinued BCG due to toxicity, compared to none in gemcitabine group. None of the toxicities were fatal.

**Table 3:** Adverse events.

Adverse events	BCG *n* (%)	Gemcitabine *n* (%)	*p*-Value
Cystitis	6 (11%)	2 (3%)	0.14
Urosepsis	5 (9%)	0	0.06
Suprapubic discomfort	3 (5%)	2 (3%)	0.66
Hematuria	5 (9%)	0	0.06
Systemic BCG infection	4 (7%)	0	0.11

Total	23 (44%)	4 (7%)	<0.05

*GEM, intravesical gemcitabine; BCG, intravesical bacillus Calmette–Guérin*.

## Discussion

Intravesical BCG has been used in the treatment of superficial bladder cancer for more than 30 years, with effectiveness demonstrated in randomized trials. Four meta-analyses have confirmed its efficacy after TURBT (13–16). However, at least 40–45% of patients have residual tumor after initial treatment and 20% of these are truly refractory (17). This situation poses a significant management dilemma with no definite guidelines available. Multiple studies have investigated various intravesical options including MMC, gemcitabine, etc. The MMC infusions have shown a response rate of 40–50%, though found to be slightly less efficacious than BCG and less well tolerated with more chemical cystitis and allergic reactions with MMC (18–20). Though epirubicin was shown to be more beneficial than TURBT alone, it was inferior to adjuvant BCG therapy in the post-TURBT setting (21).

Gemcitabine is generally not used as first-line adjuvant intravesical therapy due to the lack of clinical trials evidence comparing gemcitabine with BCG. A nationwide shortage of BCG resulted in widespread use of intravesical gemcitabine in first-line setting around 2010 in Australia. Many patients were treated with gemcitabine in the region of Canberra during this period. Clinicians noted acceptable tolerance and potential clinical benefit.

In our study, we analyzed retrospective data to compare efficacy and tolerance to BCG and gemcitabine. We noted a trend toward better DFS in gemcitabine group with a HR of 0.49 (95% CI: 0.22–1.06, *p* = 0.07, Table 2). Fifty-five percent of patients remained disease free in gemcitabine group, compared with 48% in BCG group at 2 years. It is also important to note that half of the patients in gemcitabine group had high grade cancer. Though numerically more patients benefited from intravesical gemcitabine in our study population including those with high grade NMIBC, the treatment effect was not statistically significant with multivariable analysis. The EAU risk groups and the grade of the tumor were independently associated with recurrence.

There are potential limitations in this retrospective analysis which may have impacted the outcome of the results. First, the small sample size makes the estimate of the treatment effect less robust. Second, there are some imbalance in the EAU risk groups between the two treatment groups, slightly in favor of gemcitabine which was adjusted in the multivariate model. Third, there could be other unaccounted variables such as comorbidities, which may have influenced treatment decisions. Thus, this result should be interpreted with care. This study suggests that gemcitabine could be a potentially important therapeutic option for NMIBC, in the first-line setting. It support the results of the phase 2 randomized controlled study comparing BCG and gemcitabine by Di Lorenzo et al., which showed significant improvement in DFS and lower recurrence rate with gemcitabine (11) in those who failed initial BCG therapy. A randomized controlled phase 3 trial in the first-line setting with larger number is required to clarify the place of gemcitabine therapy.

Overall patients tolerated gemcitabine well with fewer side effects than with BCG; 44% of patients experienced some form of toxicity with BCG, whereas only 7% experienced toxicity with gemcitabine (*p* ≤ 0.05). Antibiotic usage and treatment interruptions were more frequent in those who received BCG. More than 50% of patients treated with BCG had at least 1–2 interruptions whereas interruptions to treatment were minimal with gemcitabine. The observed favorable toxicity profile of intravesical gemcitabine is consistent with previous studies (3). Significantly, four patients developed systemic BCG infection necessitating systemic antituberculous treatment.

In conclusion, gemcitabine was associated with similar (with a trend toward superior) DFS and a clinically significant improved toxicity profile compared with BCG. Intravesical BCG remains the standard first-line adjuvant therapy; however, gemcitabine could be considered as a reasonable alternative for patients who are not suitable for treatment with intravesical BCG and for those who have relapsed on BCG. Gemcitabine may also be considered in elderly patients as a first-line option due to better tolerability and reduced incidence of side effects, and for those at high risk of systemic BCG infection, such as immune-compromised patients or for those with recurrent hematuria. Our findings should help stimulate a prospective phase 3 trial to confirm the benefits of first-line gemcitabine over BCG.

## Author Contributions

All authors listed have made a substantial, direct, and intellectual contribution to the work and approved it for publication.

## Conflict of Interest Statement

The authors declare that the research was conducted in the absence of any commercial or financial relationships that could be construed as a potential conflict of interest.

## Abbreviations

BCG, bacillus Calmette–Guérin; GEM, gemcitabine; NMIBC, non-muscle invasive bladder cancer; TURBT, transurethral resection of bladder tumor; MMC, mitomycin C; CIS, carcinoma *in situ*; EAU, European Association of Urology; ACT, Australian Capital Territory.
